# Indium Recovery by Adsorption on MgFe_2_O_4_ Adsorbents

**DOI:** 10.3390/ma15207054

**Published:** 2022-10-11

**Authors:** Loredana Ciocărlie, Adina Negrea, Mihaela Ciopec, Narcis Duteanu, Petru Negrea, Paula Ianasi, Catalin Ianasi, Nicoleta Sorina Nemes

**Affiliations:** 1 Faculty of Industrial Chemistry and Environmental Engineering, Polytechnic University of Timişoara, Victoriei Square, No. 2, 300006 Timişoara, Romania; 2 National Institute for Research and Development in Electrochemistry and Condensed Matter, 144th Dr. A.P. Podeanu Street, 300569 Timisoara, Romania; 3 “Coriolan Drăgulescu” Institute of Chemistry, Bv. Mihai Viteazul, No. 24, 300223 Timisoara, Romania; 4 Renewable Energy Research Institute—ICER, University Politehnica of Timisoara, 300501 Timisoara, Romania

**Keywords:** indium recovery, adsorption, iron oxide, magnesium oxide, composite

## Abstract

Indium and its compounds have many industrial applications and are widely used in the manufacture of liquid crystal displays, semiconductors, low temperature soldering, and infrared photodetectors. Indium does not have its own minerals in the Earth’s crust, and most commonly, indium is associated with the ores of zinc, lead, copper and tin. Therefore, it must be recovered as a by-product from other metallurgical processes or from secondary raw materials. The aim of this study is to investigate the adsorption properties for recovering indium from aqueous solutions using iron–magnesium composite (MgFe_2_O_4_). In addition, the results show that the material offers very efficient desorption in 15% HCl solution, being used for 10 adsorption–desorption cycle test. These results provide a simple and effective process for recovering indium. Present study was focuses on the synthesis and characterization of the material by physico-chemical methods such as: X-ray diffraction, FT-IR spectroscopy, followed by the adsorption tests. The XRD indicates that the MgFe_2_O_4_ phase was obtained, and the crystallite size was about 8 nm. New prepared adsorbent materials have a point of zero charge of 9.2. Studies have been performed to determine the influence of pH, initial indium solution concentration, material/solution contact time and temperature on the adsorption capacity of the material. Adsorption mechanism was established by kinetic, thermodynamic and equilibrium studies. At equilibrium a maximum adsorption capacity of 46.4 mg/g has been obtained. From kinetic and thermodynamic studies was proved that the studied adsorption process is homogeneous, spontaneous, endothermic and temperature dependent. Based on Weber and Morris model, we can conclude that the In (III) ions takes place at the MgFe_2_O_4_/In (III) solution–material interface.

## 1. Introduction

Indium and its compounds have widespread industrial applications in several fields [1,2]. The European Union included indium into the list of critical materials. China was reported as one the largest producers of indium in the word, reaching a total of 290 tons in 2016. For example, in 2009 about 110 tons of indium were consumed in the US, and indium consumption increases annually [1,2,3,4,5,6]. Massive development of mobile electronic devices requires a large consumption of In, used in the form of indium–tin–oxide layers into LCD construction [7]. Different estimations indicate that into the Earth’s crust indium content ranges from 50 to 200 parts per billion [8]. However, at the present consumption rate indium reserves are expected to be depleted in 20 years, and the demand and consumption of indium increases every year [9,10]. Until now, different attempts were made for In recovery from different scraps [7]. Therefore, the development of an efficient recovery process is extremely important for the stable supply of indium, along with the issue of resource recycling and environmental sustainability [11,12,13].

Conventional methods for recovering indium from secondary resources (such as industrial wastewater), include precipitation [2,14,15], solvent extraction [16], ion exchange [2,10,17,18,19,20,21], nanofiltration membranes [2,22], chemical reduction [2,23], and electroanalytical techniques [2,24]. Solvent precipitation and solvent extraction are well-known recovery methods, but they generate potential environmental problems caused by the usage of large amounts of chemicals and organic solvents [17]. However, the principal disadvantage of the solvent extraction process is represented by extractant loss, which can cause environmental hazards concomitant with economic constraints. Compared to solvent extraction, ion exchange technique is much easier. However, its low selectivity for desired metallic ions represents the main problem of such a recovery technique. Recently, the usage of impregnated resins was developed as a technological alternative for indium ion extraction [10,25,26,27,28]. Other technologies used for indium recovery from aqueous solutions are solvent extraction and resin adsorption. In order to attain a higher separation efficiency of indium from aqueous solutions it is recommended to use multi-step extraction/adsorption and reverse extraction/desorption. In addition, an increase in secondary waste production was observed when of organic solvent and acidic solutions introduction.

The pyrometallurgical method can be used for recovery of indium species, but it needs a large amount of energy to operate at high temperatures, and its ability to separate metals is not ideal. Electroplating has a high operability due to the controllable potential of the electrode and the adjustable electroreduction property of the metal by the addition of ligand [29,30] and demonstrated superiority in the extraction of metal from multimetallic waste.

Adsorption is expected to be the most suitable method for recovering indium due to its simple concept, high safety and ecological process [14,31,32,33,34,35]. In particular, different research studies were carried out in order to find a proper adsorbent material, with higher selectivity for indium ions. Such materials are represented by different polymeric resins, having grafted different functional groups [9,36]. Fortes et al. reported adsorption of indium in aqueous solution by means of chelating resin of iminodiacetic acid as a sorbent [2,37], and Tokuyama and Iwama studied solid phase extraction of indium using poly (N-isopropylacrylamide) as the sorbent [38]. The hydrogen ion of the iminodiacetic acid group on the polymeric resin could be replaced with the indium ions. In this way, indium ions are displaced and chelated inside the polymeric resin by the functional group [37]. Moreover, Calagui et al. [14], reported the adsorption of indium from aqueous solution on chitosan-coated bentonite balls. Therefore, numerous adsorptive processes have been developed for indium separation and recovery from different residual solutions. In such processes, various absorbents were used such as: starch, activated carbon, activated carbon clothing, fly ash, chitin, shrimp shell, peanut shell pellets, clay minerals, zeolites and resins [2,14,39,40,41,42,43,44,45,46].

In the present paper we describe the attempt of usage of magnesium ferrites spinel type structure as an adsorbent material for indium recovery. Until now, usage of MgFe_2_O_4_ spinel as adsorbent material has been very limited. The spinel-type structures of magnesium ferrites are of increasing interest due to their chemical and physical properties. The molecular structure of magnesium ferrites is found in the form of:

$${\left({\mathrm{Mg}}_{1-X}^{2+}{\mathrm{Fe}}_{X}^{3+}\right)}_{T}\;{\left({\mathrm{Mg}}_{X}^{2+}{\mathrm{Fe}}_{2-X}^{3+}\right)}_{O}O_{4}\;$$

Mg^2+^, Fe^3+^—divalent and trivalent cations
X—degree of inversion

In a spinel structure, cations Mg^2+^ and Fe^3+^ can occupy tetrahedral interstitial positions (T) as well as octahedron (O) of the cubic lattice formed by ions of O^2−^ [47]. Bloesser et al. shows that in order to obtain materials with the desired properties we have to change the degree of inversion by modifying the synthesis parameters [48]. If we change parameters such as temperature, the interstitial positions of magnesium ions can also change.

Hammache et al. indicate that the spinel MgFe_2_O_4_ is non-toxic to the environment [49]. From the data of the literature it was observed that the spinel MgFe_2_O_4_ presents a wide range of applications such as: photocatalysis [50], catalytic activity, gas sensor, electronics, battery anode, pigments, magnetic resonance imaging, hyperthermia therapy and targeted drug delivery.

Various methods can be used to obtain ferrite spinel such as sol-gel, pulse laser deposition, hydrothermal [49], coprecipitation [47], microemulsions, combustion metho and solid-state reaction [51]. The aim of this study is to investigate the adsorptive properties of spinel type magnesium ferrites for indium recovery from aqueous solutions.

## 2. Materials and Methods

### 2.1. Material Synthesis and Characterization

#### 2.1.1. MgFe_2_O_4_ Composite Synthesis

MgFe_2_O_4_ composite was prepared by coprecipitation method [52]. Thus, to obtain the composite adsorbent 1 g of magnesium carbonate, MgCO_3_ (SC CHIMOPAR TRADING SRL, Bucharest, Romania), 30 mL of distilled water together with 30 mL of methanol (SC CHIMOPAR TRADING SRL, Bucharest, Romania) were contacted, mixed for 1 h and then 5 mL of HNO_3_ solution (SC CHIMOPAR TRADING SRL, Bucharest, Romania) was added to reach a pH between 1.5 and 2. The HNO_3_ solution is made by adding 5 mL of concentrated HNO_3_ and 245 mL of distilled water. After another half hour, the iron (III) nitrate, Fe (NO_3_)_3_ (SC CHIMOPAR TRADING SRL, Bucharest, Romania) was added, the temperature raised to 50 °C and the solution was stirred until it was homogenous, after about 3 h. To precipitate the material at the end, ~10 mL of NaOH solution (Merck, Sigma Aldrich, Munchen, Germany) was added to increase the pH to 5. The NaOH solution was made by adding 7.5 g of NaOH beads in 100 mL of distilled water. After obtaining the precipitate, the supernatant was removed. To remove Na from the compound it was washed with excess water. The material was dried for 24 °C to 100 °C in an oven (Pol-eko model SLW 53, SDT, Rybnik, Poland), then calcined at 260 °C in an oven at a speed of 5°/min, using an oven with controlled air atmosphere (Nabertherm LHT407GN Furnaces, Lilienthal, Germany). 

#### 2.1.2. MgFe_2_O_4_ Composite Physico-Chemical Characterization

##### Thermogravimetric Analysis, DTG

The differential thermal analysis, DTG, was performed to highlight the temperature dependence of the physical properties, using a TGA/SDTA 851-LF Mettler-Toledo. The decomposition was performed in the presence of air and the sample was heat treated in the range of 25–900 °C.

##### Fourier Transform Infra-Red Spectroscopy, FT-IR

The material was characterized by Fourier transform infrared spectroscopy (FT-IR) by using a JASCO FT/IR-4200 apparatus (SpectraLab, Shimadzu, Japan).

##### X-ray Diffraction Analysis, XRD

In order to obtain information about the degree of crystallinity of the material and the presence of several phases in the material, X-ray diffraction analysis was performed, XRD (D8 Advance-Bruker AXS), using Mo-Kα radiation (αMo = 0.7093 Å).

##### pH_pZc_

Point of zero charge, pHpZc, was determined by bringing the studied system to equilibrium. In this case, 0.1 g of adsorbent material (MgFe_2_O_4_) wZ mixed with 25 mL of 0.1 N KCl solution at 200 rpm and a temperature of 298 K, using a water bath with thermostating and stirring, Julabo SW23 type. KCl solutions pH was modified in range 2–12, by adding NaOH solutions with a concentration between 0.05 N and 2 N or HNO_3_ solutions with a concentration between 0.05 N and 2 N. Further, all the samples were filtered and subsequently was determined the pH of the resulting solution by using a pH meter (METTLER TOLEDO, SevenCompact, S 210 type).

#### 2.1.3. Adsorption Studies

##### pH Effect

In the present paper we studied the influence of pH on the adsorption process of In (III) on the synthesized material, varying the pH, in the range 1–14. Thus, 0.1 g of material was kept in contact with 25 mL of In (III) solution (InCl_3_, 99.995% purity, ACROS organics, India) of initial concentration, C_0_ = 100 µg L^−1^, for 60 min in a JUABO type thermostatic bath (SW 23), at a temperature of 298 K. The pH of the solutions was adjusted using HNO_3_ and NaOH solutions having concentrations in the range of 0.1–1 N, obtained by diluting 63% HNO_3_ (Carl Roth) and NaOH, pellets (Merck Sigma Aldrich).

##### Contact Time and Temperature Effect

In order to establish the influence of contact time and temperature on material adsorption capacity, 0.1 g of adsorbent material were weighed and mixed with a 25 mL solution containing 100 µg L^−1^ In (III). Different samples were prepared, which were mixed for different times (15, 30, 60 and 120 min), at different temperatures (298, 308, 318 and 328 K) and 200 rpm using a thermostatic bath.

##### Initial Concentration Effect

In order to establish the effect of the initial concentration of In (III) ions on the adsorption capacity of the MgFe_2_O_4_ adsorbent material, solutions with concentrations of 5 × 10^2^, 1 × 10^3^, 1,5 × 10^3^, 2 × 10^3^, 3 × 10^3^, 5 × 10^3^, 7 × 10^3^, 8 × 10^3^, 1 × 10^4^, 5 × 10^4^, 1 × 10^5^, 2 × 10^5^ and 3 × 10^5^ µg L^−1^ were prepared. These solutions were prepared by appropriate dilution from a stock solution of InCl_3_, 1000 mg L^−1^. All adsorption tests were conducted at pH > 2 for 90 min and at 298 K. In order to evaluate the adsorption capacity was measured the residual concentration of In (III), by using the graphite furnace atomic absorption spectrophotometer (AA 6800, Schimadzu).

##### Kinetics Adsorption

In order to study the adsorption kinetic for studied adsorption process obtained experimental data were modelled with three different kinetic models: Lagergren, Ho and McKay, and Weber and Morris one. Equations used to describe adsorption kinetic are presented in Table 1.

##### Adsorption Isotherm Models

Similar, in order to better understand and describe the adsorption equilibrium, experimental data were modelled using Langmuir, Freundlich and Sips isotherms. Equations used to describe adsorption equilibrium are presented in Table 2.

##### Thermodynamic Studies

The amount of activation energy, Ea, provides information about the nature of the adsorption process whether it is physical or chemical. Further, starting from the experimental data obtained for In (III) adsorption on the prepared adsorbent, we evaluated the activation energy based on the Arrhenius equation:

(8) $$\ln\;k_{2}=\mathrm{lnA}-\frac{E_{a}}{\mathrm{RT}}$$

where:

k_2_—speed constant (g min^−1^ mg^−1^)
A—Arrhenius constant (g min mg^−1^)
E_a_—activation energy (kJ mol^−1^)
T—absolute temperature (K)
R—ideal gas constant (8.314 J mol^−1^ k^−1^).

Taking in account that the pseudo-second-order model is better fitting obtained experimental data, the activation energy was calculated by using the speed constant obtained based on this model. Based on the slope of the linear dependence lnk_2_ versus 1/T, was determined the value of activation energy.

Further, based on Gibbs–Helmholtz equation was calculated the value of Gibbs energy, used to establish if the In (III) adsorption onto the prepared adsorbent material is a spontaneous process [33]:

(9) $${\Delta G}^{{}^{\circ}}={\Delta H}^{{}^{\circ}}-T\cdot {\Delta S}^{{}^{\circ}}$$

where:

ΔG^°^—free Gibbs energy standard variation (J mol^−1^)
ΔH^°^—enthalpy standard variation (J mol^−1^)
ΔS^°^—entropy standard variation (J mol^−1^ k^−1^)
T—absolute temperature (K)

Standard variations of enthalpy and entropy were evaluated from linear dependence of ln K_d_ versus 1/T (linear form of van’t Hoff equation), where K_d_ is the equilibrium constant, which was calculated as ratio between equilibrium adsorption capacity (q_e_) and equilibrium concentration (C_e_).

## 3. Results and Discussion

### 3.1. Material Synthesis and Characterization

#### 3.1.1. Thermogravimetric Analysis, DTG 

**Figure 1:**
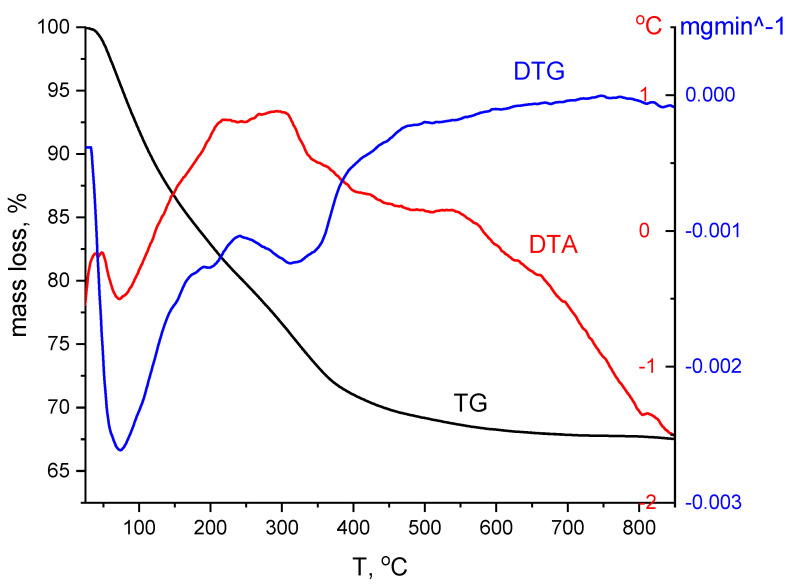
Thermogravimetric analysis for MgFe_2_O_4_ material.

Figure 1 shows that the material decomposes in three steps. In the first part of the process up to 200 °C there are two specific peaks associated with elimination of water and organic solvents with a mass loss of 16.35%. In the second part of the process an exothermic process takes place, attributed to iron nitrate III beginning to decompose, and NOx is formed. At the same time, the decomposition of MgCO_3_ and CO_2_ takes place [60]. 

At this stage in the range of 200–500 °C there is a loss of 14.49% with the formation of the spinel MgFe_2_O_4_ and partially a crystalline phase of Fe_3_O_4_. In the last stage, the material has a loss of about 1.78%. At a temperature of 875 °C, the material has a plateau meaning that most of the processes have taken place.

Chemical analysis was performed after calcination of the sample up to la 900 °C indicating that Mg and Fe ions are in ideal proportions for the formation of MgFe_2_O_4_.

#### 3.1.2. X-ray Diffraction Analysis, XRD

**Figure 2:**
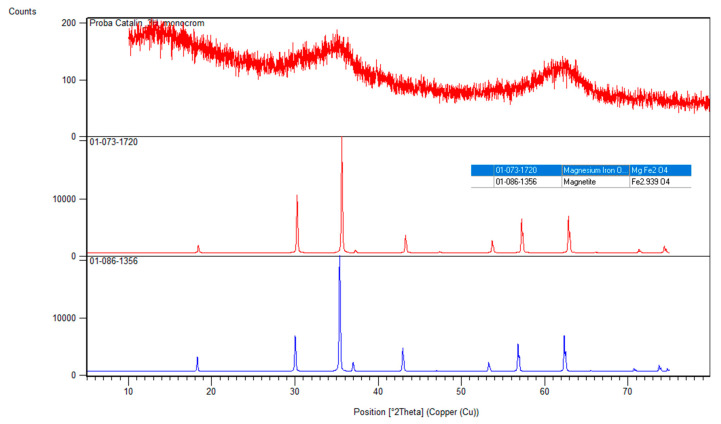
XRD MgFe_2_O_4_ material treated at 260 °C.

Figure 2 shows X-ray diffractograms for MgFe_2_O_4_ powder calcined at 260 °C.

The general appearance of the material is that it is amorphous, but following the peaks from 30, 35, 57 and 63, 2θ, which are associated with the presence of the crystalline phases 220, 311, 333 and 440, can conclude that a MgFe_2_O_4_ phase was obtained. The material corresponds to a cubic-type network, Fd-3m. The sampled data were evaluated using the reference sheet 01-073-1720 indicating that the major phase MgFe_2_O_4_. A secondary phase is formed to a small extent being specific to magnetite. Using Scherrer’s equation, the crystallite size was determined using the peaks at 311 and 440 with an average value of about 8 nm.

#### 3.1.3. FT-IR Spectra

**Figure 3:**
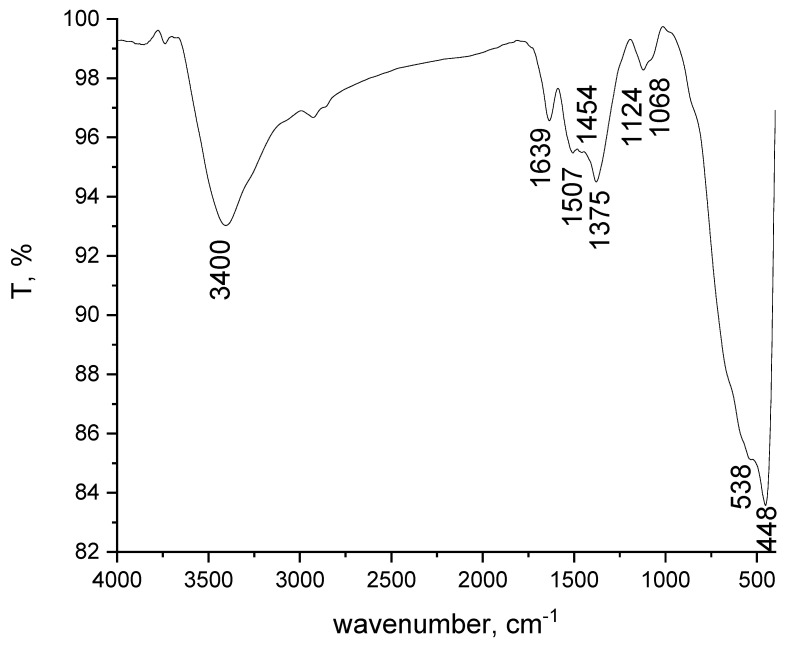
FT-IR spectra of the MgFe_2_O_4_ composite.

Figure 3 shows the FT-IR spectrum of the MgFe_2_O_4_ material, recorded into the range 4000 and 400 cm^−1^.

By analysing the spectrum depicted in Figure 3 the presence of the specific bands for -OH stretching and bending vibrations are observed, located at 3400 and 1639 cm^−1^, respectively [47]. The 1507, 1454 and 1375 cm^−1^ bands specific for stretching vibration of carbon groups are also present in the spectrum. The adsorption band at 1124 and 1068 cm^−1^ could be attributed to the presence of nitrates ion [61]. 

The most important bands obtained at 538 and 448 cm^−1^ are specific for the formation of the spinel ferrite structure. Referring to the literature, we note that in the peak located at 448 cm^−1^, it is specific for octahedral Mg-O and Fe-O. In our case, the peak at 538 cm^−1^ shows the clear formation of spinel MgFe_2_O_4_ or/and tetrahedral Fe-O [62,63]. 

These absorbance bands are attributed to vibration of tetrahedral (higher energy band of M-O) and octahedral (lower frequency band of M-O) complexes, respectively [64,65]. 

#### 3.1.4. Point of Zero Charge (pH_pZc_) Determination 

**Figure 4:**
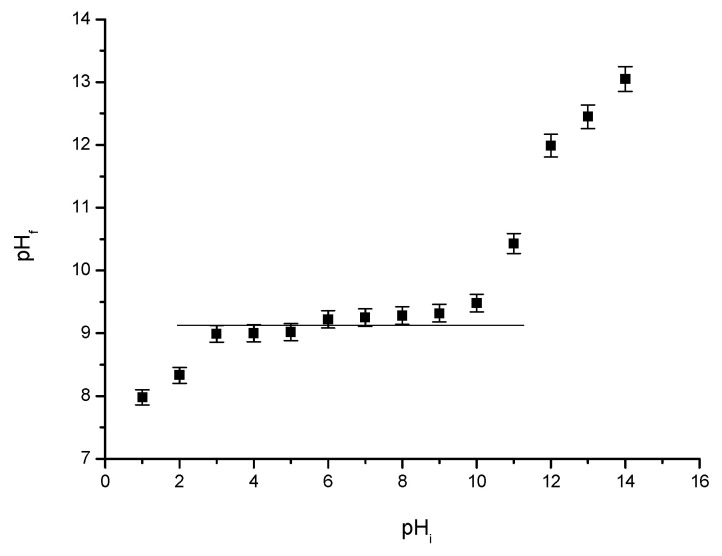
pH_pZc_ for MgFe_2_O_4_ material treated at 260 °C.

The value of point of zero charge (pHpZc) was determined using the so-called method “11 points” [66], which was also used by Freitas et al. in their studies [34]. pH_pZc_ is defined as the value where the final pH remains constant [34,67]. The pZc is determined using the graphical representation of the initial pH vs. final pH (Figure 4). 

In our case the pH_pZc_ is 9.2. This is considered to be the ideal working pH. pZc value represents a clear indication of whether the adsorbent material surface is positively or negatively charge, and depends on the pH value [67].

### 3.2. Adsorption Studies

#### 3.2.1. pH Effect

**Figure 5:**
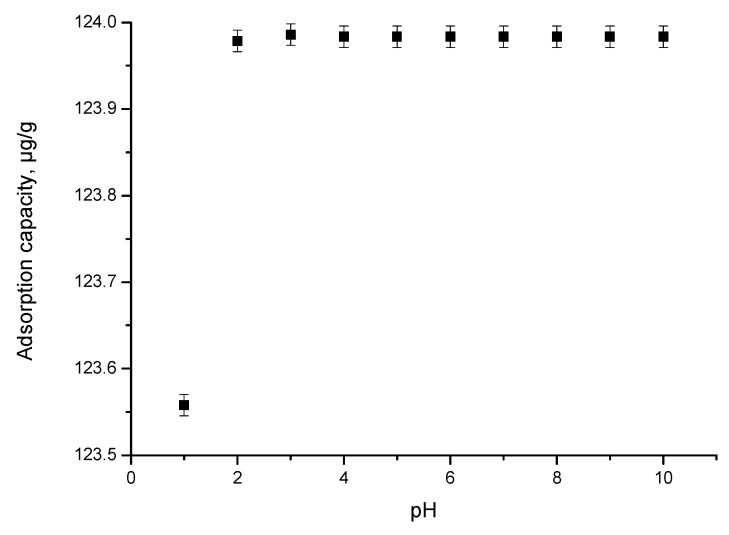
pH effect.

The pH effect of the In (III) adsorption is shown in Figure 5.

It is observed that at pH < 2, the adsorption capacity increases with the pH increase, reaching a maximum value of 124 µg In (III)/g MgFe_2_O_4_ at pH 2. By any further increase in the pH value, one can observe that the adsorption capacity remains constant. At a pH value lower than 2, according to the literature data, specific species of In (III) coexisting in solution can be: $\mathrm{In}{\left(\mathrm{OH}\right)}_{3}^{0};\;\mathrm{In}{\left(\mathrm{OH}\right)}_{2}^{+}\;\mathrm{or}\;\mathrm{In}{\left(\mathrm{OH}\right)}_{4}^{-}$ [68,69].

#### 3.2.2. Contact Time and Temperature Effect

**Figure 6:**
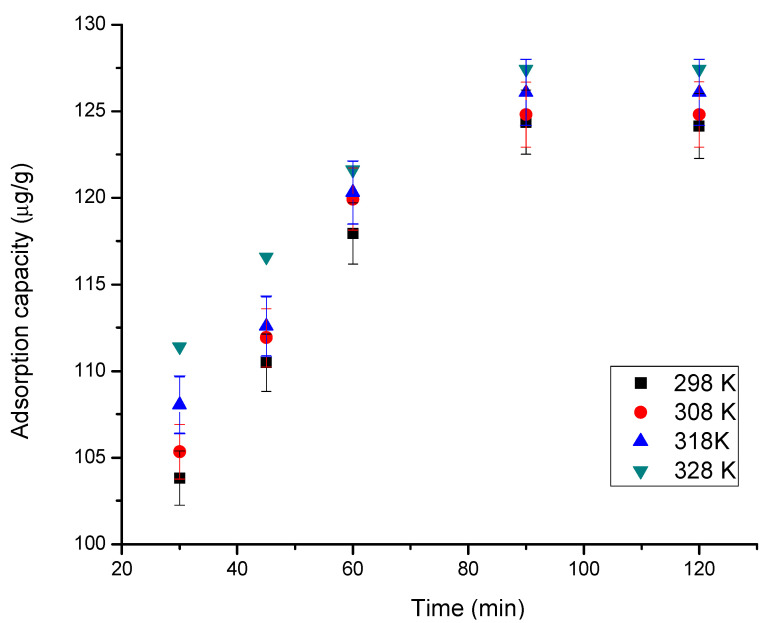
Contact time and temperature effect.

The effect of contact time (between 15–120 min) at four different temperatures (298, 308, 318 and 328 K) was studied (obtained data are depicted in Figure 6). 

The results obtained indicate that by increasing the contact time, MgFe_2_O_4_ adsorption capacity increases. Moreover, it can be observed that the constant adsorption capacity is reached (~125 µg In (III)/g) after 90 min [70]. With increasing temperature, the adsorption capacity increases, but insignificantly, which is why subsequent studies are performed only at 298 K.

#### 3.2.3. Kinetic Studies

**Figure 7:**
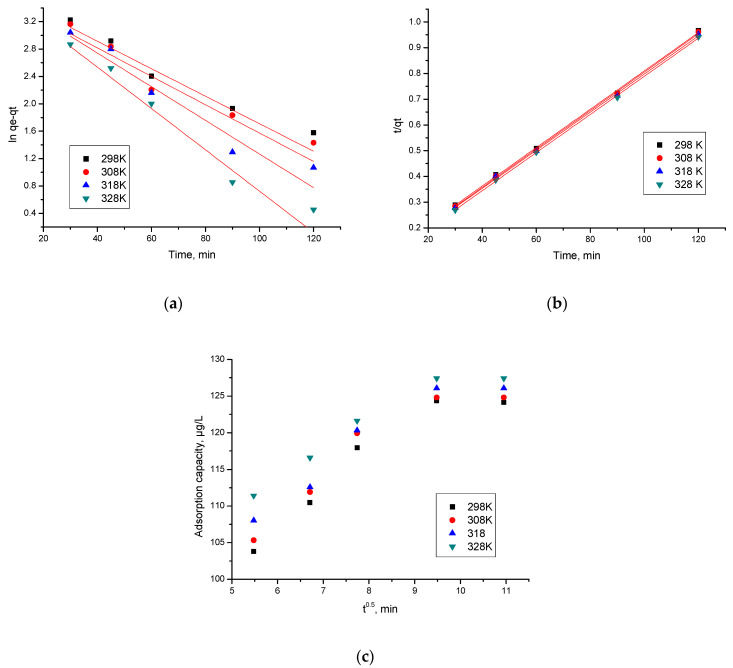
Kinetic studies. (**a**) Pseudo-first order; (**b**) pseudo-second order; (**c**) intraparticle diffusion.

Kinetics of In (III) adsorption on MgFe_2_O_4_ material was also evaluated. For that, obtained experimental data were modelled using the equations of the pseudo-first-order and pseudo-second-order kinetic models (Figure 7a,b). To distinguish whether film diffusion or intraparticle diffusion it is the speed determinant step, obtained experimental data were modelled according to the Weber and Morris model, studying intraparticle diffusion (Figure 7c).

Based on the results obtained from modelling, were evaluated the values of the speed constants, as well as the values obtained for the diffusion coefficient and C parameters, data presented in Table 3. Withal were calculated the values of the regression coefficient, R^2^ (depicted in same table).

From the data depicted in Table 3, it can be seen that obtained experimental data are modelled well by the pseudo-second-order kinetic model, proved by the regression coefficient value closer to one, R^2^~1 (0.9991–0.9996). When obtained experimental data were modelled according to the pseudo-first-order kinetic model one, R^2^ is between 0.8930 and 0.9114. Moreover, the value of q_e,calc_ calculated based on the pseudo-second-order isotherm it is close to the experimental one (q_e, exp_). Values of the calculated parameters (k_2_, q_e,calc_) are influenced by the temperature value, but not significantly, so it is not necessary to work at temperatures higher than 298 K.

At the same time, it is observed that the adsorption mechanism of In (III) is taking place in several stages, because the line obtained by the graphical representation of the dependence of q_t_ = f(t^1/2^) at different temperatures, are not passing through the origin (C = 0). Thus, we can say that both intraparticle diffusion and film diffusion influence the kinetics of adsorption. From the data presented in Table 4 it is observed that with increasing temperature the K_diff_ value also increases. It is also observed that, specific to stage 1, the diffusion constants are higher than the diffusion constants specific to stage 2, which allows us to state that the determinant of velocity is stage 1 and that in stage 2 the process is slower [71]. 

#### 3.2.4. Activation Energy

**Figure 8:**
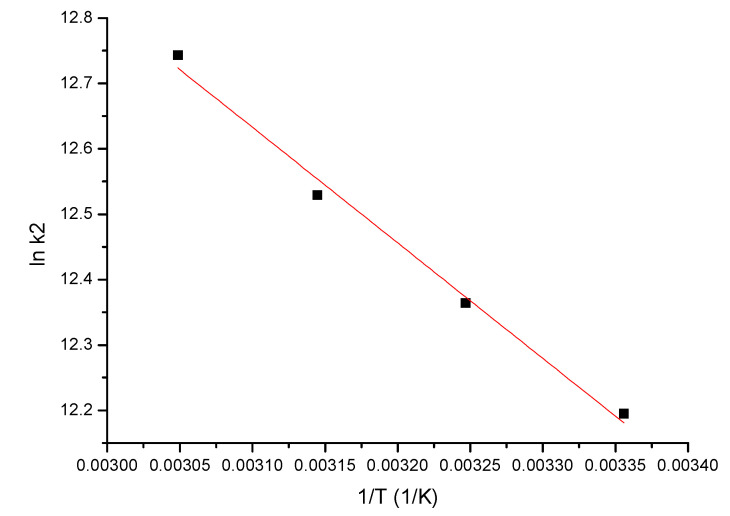
lnk_2_ vs. 1/T.

The value of the activation energy, *E_a_*, offers information about the nature of the adsorption process, whether it is a physical or chemical one. This is calculated based on graphical representation of lnK_2_ vs. 1/T, based on Arrhenius’ equation (Figure 8). In the case of the studied process (In (III) adsorption on the MgFe_2_O_4_ material), the activation energy *E_a_* was calculated, using the rate constant from the pseudo-second-order kinetic model *k_2_*.

Based on linear dependence depicted in Figure 8, it was calculated the activation energy value (7.46 kJ/mol), which was below 40 kJ/mol, shows us that the studied adsorption process is a physical one [72]. 

#### 3.2.5. Equilibrium Studies

**Figure 9:**
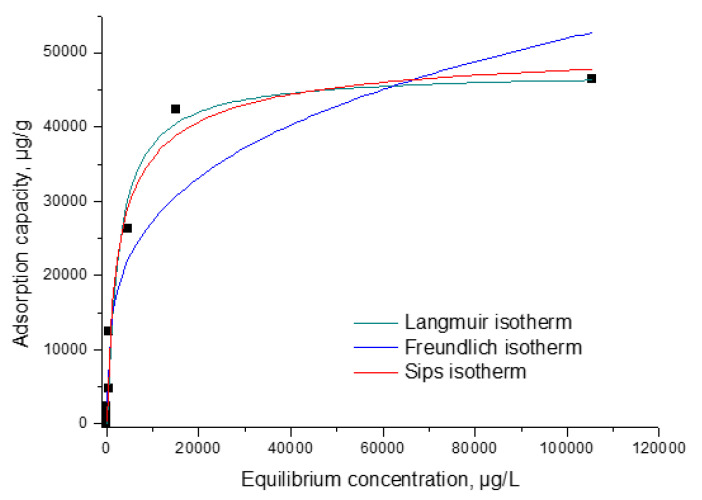
Equilibrium studies.

The adsorption mechanism was established by modelling experimental data with three different isotherms: Langmuir, Freundlich and Sips (obtained data being presented in Figure 9). Isotherms are applied to model obtained experimental data to determine the maximum adsorption capacity of the material. The Freundlich isotherm model assumes that the surface area of the material with adsorbent properties is heterogeneous, so it can be considered that the heat distribution required for the adsorption process on the surface of the adsorbent material is uneven and multilayer adsorption can occur due to unlimited active centres. The Sips isotherm is derived from the Langmuir and Freundlich isotherms. In the case of low adsorbate concentrations, it is reduced to the Freundlich isotherm and, if the adsorbate concentrations are high, it has the characteristics of the Langmuir isotherm. Therefore, this isotherm can be used to calculate the adsorption capacity.

From the data presented in Figure 9 were evaluated the specific parameters associated with each isotherm used for modelling the experimental, parameters depicted in Table 4.

The relationship between the equilibrium concentration (C_e_) of In (III) and the adsorption capacity demonstrates that as the equilibrium concentration increases, so does the adsorption capacity until equilibrium is reached, establishing the maximum adsorption capacity obtained experimentally, q_e,exp_ (~46.4 mg In (III)/g). It was found, according to the data in Table 5, that the model that best describes the adsorption process is the Sips one, because the regression coefficient, R^2^, is closest to 1 (R^2^ = 0.9877), and the theoretical adsorption capacity ~47.5 mg In (III)/g is near the experimental one. Taking in account the data presented in the literature, a comparison of the adsorption capacity obtained for the new prepared material for the recovery of In (III) with adsorption capacity obtained for other materials is presented in Table 5. Based on presented data it was found that the material MgFe_2_O_4_ present highest adsorption capacity.

#### 3.2.6. Thermodynamic Studies

**Figure 10:**
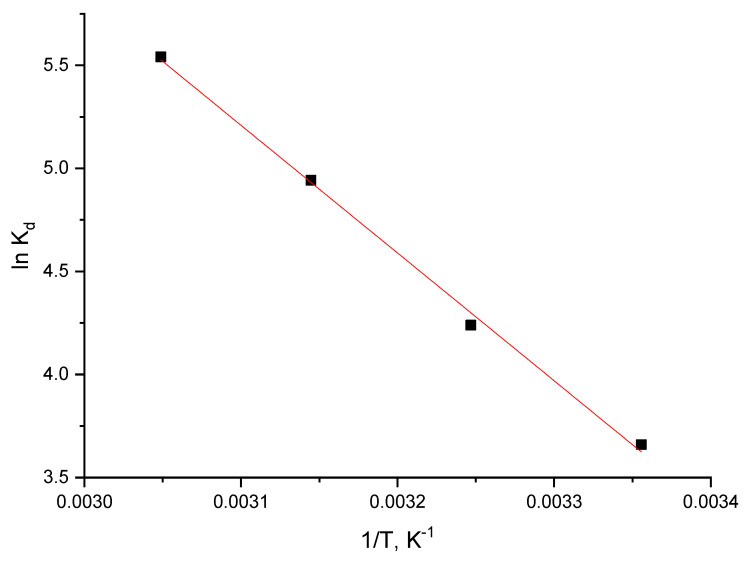
Thermodynamic studies.

In order to evaluate the value of Gibbs free energy were performed thermodynamic studies in the temperature range 298–328K, by using the Gibbs–Helmholtz equation. Based on the van’t Hoff equation and from the equation of the line obtained from the graphical representation of ln *K_d_* = f(1/*T*), according to Figure 10, one can calculate the standard variation of the entropy ΔS° and the standard variation of the enthalpy ΔH°. 

Table 6 shows the thermodynamic parameters resulting from the three temperatures.

From the resulting data, it is observed that ΔH^°^ has a positive value, meaning that the studied adsorption is endothermic. It is also observed that ΔG^°^ has negative values, increasing in absolute value with temperature increase, indicating that the adsorption process is spontaneous and influenced by temperature. The value of ΔS^°^ is positive which indicates that the adsorption process is favoured, flowing at the interface of the material MgFe_2_O_4_/solution with In (III).

An important aspect is that it is represented by the ability to reuse the new adsorbent material. After indium adsorption, the exhausted material was treated with 15% HCl in order to regenerate it. In this way, spinel type adsorbent material was reused 11 times. 

## 4. Conclusions

Obtained experimental data prove that the new prepared adsorbent material (MgFe_2_O_4_) can be used with good results for the recovery by adsorption of indium from aqueous solutions. The MgFe_2_O_4_ composite was synthesized by the sol-gel method and further characterized by thermogravimetric analysis, Fourier transform infrared spectroscopy and X-ray analysis. The purpose of these analyses was to highlight the formation by heat treatment of magnesium ferrite, MgFe_2_O_4_, predominantly and as a secondary phase magnetite. Based on experimental data obtained after performing these analyses we can conclude that the new prepared adsorbent material is preponderantly represented by magnesium ferrite. Further, MgFe_2_O_4_ was used in the adsorption experiment in order to recover In ions from aqueous solutions. As a result of the adsorption experiment, we established the working conditions needed to obtain the best adsorption capacity of the material used for In (III) recovery from aqueous solutions. In this context, optimum conditions are: pH > 2, contact time 90 min, temperature 298 K, initial concentration 200 mg L^−1^. By conducting the adsorption process in optimal conditions we obtained a maximum adsorption capacity of 46.4 mg of In (III) per each gram of adsorbent material. The mechanism of the adsorption process has been highlighted by kinetic, thermodynamic and equilibrium studies. Taking into account the obtained experimental data we can conclude that the studied adsorption is homogeneous, spontaneous, endothermic and temperature-dependent. Based on the Weber and Morris model we can conclude that the In (III) ions takes place at the MgFe_2_O_4_/In (III) solution–material interface.

## Figures and Tables

**Table 1:** Equations used to describe adsorption kinetics.

Parameters	Equation	References
Pseudo-first order kinetic model (Lagergren)	$\ln\;\left(q_{e}-q_{t}\right)={\mathrm{lnq}}_{e}-k_{1}\;t$ (1) where: q_e_—equilibrium adsorption capacity (mg g^−1^) q_t_—adsorption capacity at a specific time—t (mg g^−1^) k_1_—pseudo-first order speed constant (min^−1^) t—contact time (min)	[53]
Pseudo-second order kinetic model (Ho and McKay)	$\frac{t}{q_{t}}=\frac{1}{k_{2}\;q_{e}^{2}}+\frac{t}{q_{e}}$ (2) q_e_—equilibrium absorption capacity (mg g^−1^) q_t_—adsorption capacity at a specific time—t (mg g^−1^) k_2_—pseudo-second order speed constant (g mg^−1^ min^−1^) t—contact time (min)	[54,55]
Intraparticle diffusion (Weber and Morris model)	*Q_t_* = *k_diff_* • *t*^1/2^ + *C* (3) where: *Q_t_*—adsorption capacity at t time, µg/g *k_diff_*—speed constant for intraparticle diffusion, µg/g·min^1/2^ *C*—constant correlated with the thickness of the liquid film surrounding the adsorbent particles.	[56,57]

**Table 2:** Equations used to describe adsorption equilibrium.

Parameters	Equation	References
The adsorption capacity of the material	$q_{e}=\frac{\left(C_{0}-C_{e}\right)\;\;V}{m}$ (4) where: q_e_—the maximum adsorption capacity (µg g^−1^) C_0_—initial concentration of metallic ion in solution (µg L^−1^) C_e_—the equilibrium concentration of metallic ion in solution (µg L^−1^) V—volume of the aqueous solution with metallic ion content (L) m—mass of the adsorbent (g)	
Langmuir isotherm nonlinear expression	$q_{e}=\frac{q_{L}\;K_{L}\;C_{e}}{1+K_{L}\;C_{e}}$ (5) where: q_e_—the maximum adsorption capacity (µg g^−1^) C_e_ the equilibrium concentration of metallic ion in solution (µg L^−1^) q_L_—Langmuir maximum adsorption capacity (µg g^−1^) K_L_—Langmuir constant	[58]
Freundlich isotherm nonlinear expression	$q_{e}=K_{F}\;C_{e}^{1/n_{f}}$ (6) where: q_e_—the maximum adsorption capacity (µg g^−1^) C_e_—the equilibrium concentration of metallic ion in solution (µg g^−1^) K_F_ and n_F_—the characteristic constants that can be related to the relative adsorption capacity of the adsorbent and the intensity of adsorption	[35]
Sips isotherm nonlinear expression	$q_{e}=\frac{q_{s}\;K_{S}\;C_{e}^{1/n_{S}}}{1+K_{S}\;C_{e}^{1/n_{S}}}$ (7) where: q_S_—the maximum adsorption capacity (µg g^−1^) K_S_—constant related to the adsorption capacity of the adsorbent n_S_—the heterogeneity factor	[59]

**Table 3:** Kinetic parameters for the adsorption of In (III) onto MgFe_2_O_4_.

**Pseudo-First-Order**				
**Temperature (K)**	***q*** **_e,exp_** **(µg g^−1^)**	***k*** **_1_** **(min^−1^)**	***q*** **_e,calc_** **(µg g^−1^)**	**R^2^**
298	124.3	0.020	39.61	0.8930
308	125.0	0.022	41.15	0.8883
318	126.0	0.023	41.63	0.9063
328	127.4	0.030	42.17	0.9114
**Pseudo-second-order**				
**Temperature (K)**	***q*** **_e,exp_** **(µg g^−1^)**	***k*** **_2_** **(g µg^−1^∙min^−1^)**	***q*** **_e,calc_** **(µg g^−1^)**	**R^2^**
298	124.3	197.7	113.6	0.9991
308	125.0	234.2	119.0	0.9992
318	126.0	276.2	126.5	0.9992
328	127.4	342.4	128.2	0.9996
**Intraparticle diffusion model (IPD)**				
**Temperature (K)**	**K_diff_ (µg·g^−1^ min^−1/2^)**	**C**		**R^2^**
298	3.06	84.7		0.8646
308	3.54	87.7		0.8370
318	3.67	90.0		0.8721
328	3.88	96.1		0.8646

**Table 4:** Parameters of isotherm model for adsorption In (III) onto MgFe_2_O_4_.

**Langmuir Isotherm**			
**q** **_m,exp_** **(µg g^−1^)**	**K** **_L_** **(L µg^−1^)**	**q** **_L_** **(µg g^−1^)**	**R** **^2^**
46,396	3.84 × 10^−4^	51,616	0.9717
**Freundlich isotherm**			
**K** **_F_** **(µg g^−1^)**	**1/n_F_**		**R** **^2^**
2107.4	0.28		0.9049
**Sips isotherm**			
**K** **_S_**	**q** **_S_** **(µg/g)**	**1/n_S_**	**R** **^2^**
0.28	47,475	0.03	0.9877

**Table 5:** Comparison of adsorption performance with other material for In (III) adsorption.

Materials	q, mg/g	References
Cellulose with iminodiacetic acid	0.172	[70]
CCB	7.89	[14]
Amberlite IRA-400AR	14.93	[73]
Coated solvent impregnated resins	23.8	[74]
Modified solvent impregnated resins	26.25	[10]
Dowex 1	31.00	[73]
Modified solvent impregnated resin (MSIR—impregnation of sec-octylphenoxy acetic acid (CA-12) on styrene–divinylbenzene copolymer support (HZ818) after nitration of the benzene rings)	26.0	[10]
Non-imprinted polymers In(III)-NIP	29.75	[2]
LewatitTP 260	1.437	[7]
Lewatit TP 208	1.488	[7]
Amberlite IRA 743	0.84	[7]
MgFe_2_O_4_	~46.4	This paper

**Table 6:** Thermodynamic parameters for adsorption of In (III) onto MgFe_2_O_4_.

ΔH^°^ (J mol^−1^)	ΔS^°^ (J mol^−1^ K^−1^)	ΔG^°^ (kJ mol^−1^)				R^2^
51.52	203.0	298 K	308 K	318 K	328 K	0.9975
		−60.44	−62.47	−64.50	−66.53	
